# Blockade of the CXCR3/CXCL10 axis ameliorates inflammation caused by immunoproteasome dysfunction

**DOI:** 10.1172/jci.insight.152681

**Published:** 2022-04-08

**Authors:** Yuki Sasaki, Hideki Arimochi, Kunihiro Otsuka, Hiroyuki Kondo, Shin-ichi Tsukumo, Koji Yasutomo

**Affiliations:** 1 Department of Immunology and Parasitology, Graduate School of Medicine,; 2 Department of Interdisciplinary Research for Medicine and Photonics, Institute of Post-LED Photonics, Tokushima, and; 3 The Research Cluster Program on Immunological Diseases, Tokushima University, Tokushima, Japan.

**Keywords:** Genetics, Inflammation, Ubiquitin-proteosome system

## Abstract

Immunoproteasomes regulate the degradation of ubiquitin-coupled proteins and generate peptides that are preferentially presented by MHC class I. Mutations in immunoproteasome subunits lead to immunoproteasome dysfunction, which causes proteasome-associated autoinflammatory syndromes (PRAAS) characterized by nodular erythema and partial lipodystrophy. It remains unclear, however, how immunoproteasome dysfunction leads to inflammatory symptoms. Here, we established mice harboring a mutation in *Psmb8* (Psmb8-KI mice) and addressed this question. Psmb8-KI mice showed higher susceptibility to imiquimod-induced skin inflammation (IMS). Blockade of IL-6 or TNF-α partially suppressed IMS in both control and Psmb8-KI mice, but there was still more residual inflammation in the Psmb8-KI mice than in the control mice. DNA microarray analysis showed that treatment of J774 cells with proteasome inhibitors increased the expression of the *Cxcl9* and *Cxcl10* genes. Deficiency in *Cxcr3*, the gene encoding the receptor of CXCL9 and CXCL10, in control mice did not change IMS susceptibility, while deficiency in Cxcr3 in Psmb8-KI mice ameliorated IMS. Taken together, these findings demonstrate that this mutation in *Psmb8* leads to hyperactivation of the CXCR3 pathway, which is responsible for the increased susceptibility of Psmb8-KI mice to IMS. These data suggest the CXCR3/CXCL10 axis as a new molecular target for treating PRAAS.

## Graphical abstract

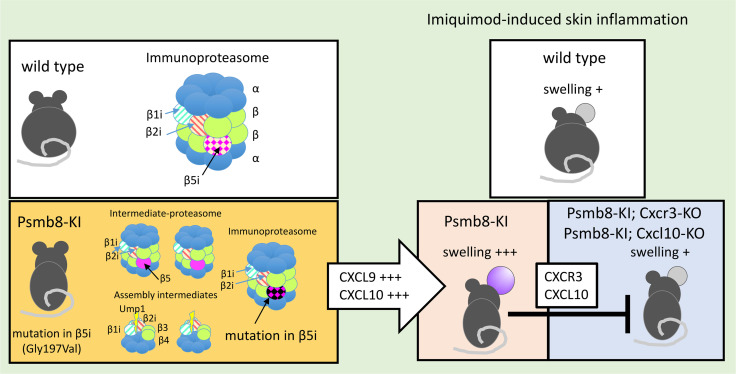

## Introduction

Proteasomes degrade ubiquitin-coupled proteins in the cytoplasm and nucleus and are crucial for various types of cellular regulation (1–4). The 26S proteasome is composed of a 19S regulator and 20S proteolytic core complex, and the 19S regulator acts as an ubiquitin receptor with an ATPase ring that regulates protein unfolding. The 20S core complex has 4 rings with 7 subunits. Immunoproteasomes were initially identified as IFN-γ–inducible proteasomes and characterized by preferential cleavage of ubiquitinated proteins to generate potential T cell epitopes that bind to MHC class I (4–6). Three inducible β subunits of the immunoproteasome are low molecular mass polypeptide 2 (LMP2; β1i), multicatalytic endopeptidase complex-like 1 (MECL-1; β2i), and LMP7 (β5i). The corresponding constitutive subunits β1, β2, and β5 are replaced by β1i, β2i, and β5i. These exchanges from constitutive subunits to immunoproteasome subunits change the cleavage specificity; in immunoproteasomes, caspase-like activity is strongly reduced, and chymotrypsin-like activity is enhanced. The preference for MHC class I binding is caused by a selective enhancement of chymotrypsin-like activity and the unique structural character, which enhances the generation of peptides with C-terminal hydrophobic and basic amino acids; thus, these peptides fit well in the groove of MHC class I (7).

Various studies have demonstrated the roles of immunoproteasomes in cellular differentiation and disease progression by using proteasome inhibitors and genetically modified mice (2, 4). Although mice genetically deficient in one of the catalytic subunits of immunoproteasomes, such as β1i, β2i, or β5i, have been used in many studies (8–10), mice deficient in a single gene or even 3 genes in the absence of thymic proteasomes did not show any inflammatory phenotype (11). We and other groups identified *PMSB8* with a missense mutation as the causative gene of Japanese autoinflammatory syndrome with lipodystrophy; Nakajo-Nishimura syndrome; chronic atypical neutrophilic dermatosis with lipodystrophy, elevated temperature syndrome, and joint contractures; muscular atrophy; microcytic anemia; and panniculitis-induced lipodystrophy syndrome, which are characterized by persistent inflammation in adipose tissue, progressive lipodystrophy, splenomegaly, and hypergammaglobulinemia without an immunodeficient phenotype (12–14). A subsequent study showed that mutations in other subunits of immunoproteasomes cause a similar syndrome (15). Collectively, these syndromes are now named proteasome*-*associated autoinflammatory syndromes (PRAAS). A mutation in an immunoproteasome subunit disturbs proteosome assembly, which results in dysfunctional proteasome activity (12). The identification of human patients with immunoproteasome dysfunction provided insight for not only the involvement of immunoproteasomes in human diseases, but also the roles of immunoproteasomes in various cellular regulatory processes.

Initially, activation of the p38 pathway was reported in patients with PRAAS (12, 14). Subsequently, an interferon signature was reported, and treatment of patients with PRAAS with a JAK1/2 inhibitor was shown to at least partially ameliorate their inflammatory symptoms (16). However, it remains unclear how immunoproteasome dysfunction causes inflammation and which molecules are key in the inflammation in patients with PRAAS. In the present study, we sought to identify key molecules that initiate or enhance inflammatory responses induced by immunoproteasome dysfunction. To do this, we established mice in which the *Psmb8* gene contains a mutation found in patients with PRAAS. We found that the CXCR3 pathway is activated in an imiquimod-induced skin inflammation (IMS) model. Blockade of the CXCR3 pathway ameliorated the inflammatory responses caused by immunoproteasome dysfunction, suggesting that the CXCR3 pathway could be a drug target in patients with PRAAS.

## Results

### Establishment of Psmb8-KI mice.

**Figure 1:**
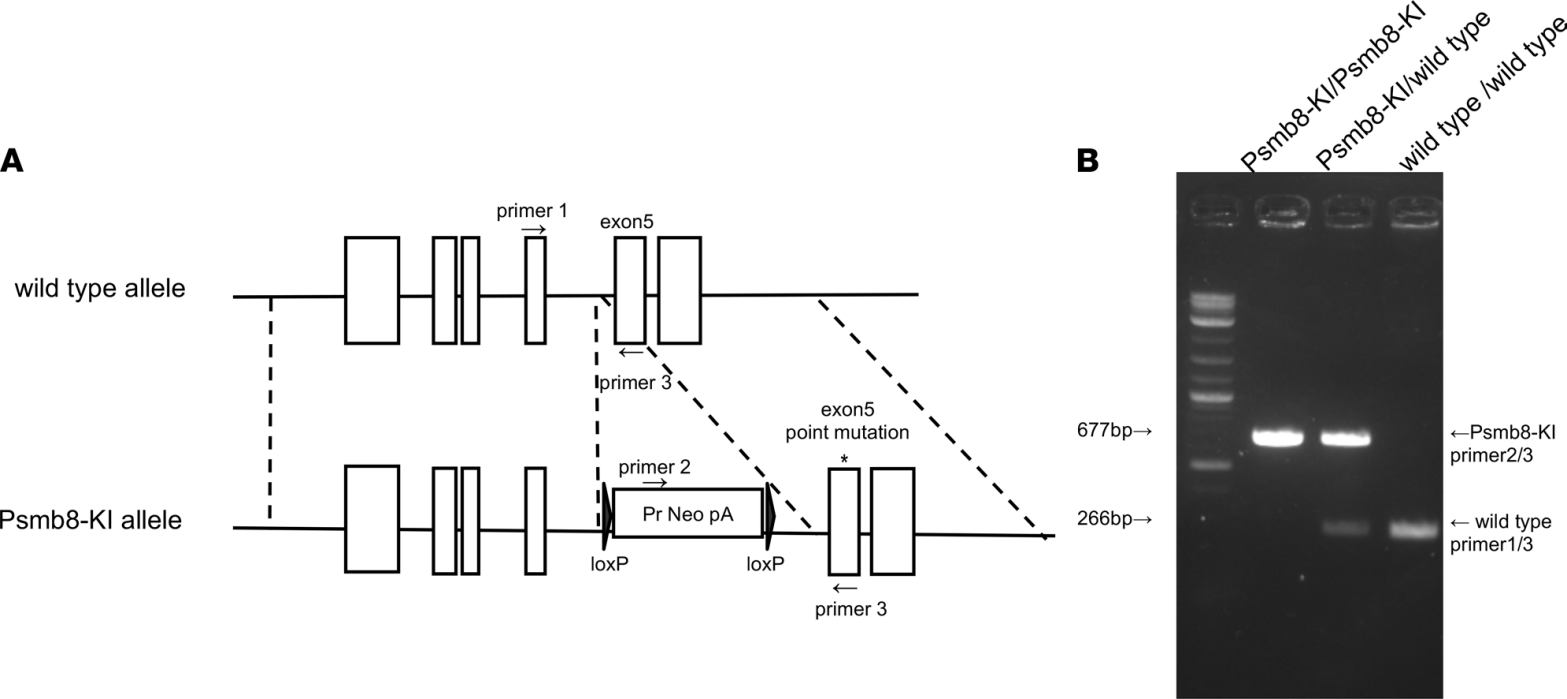
Establishment of Psmb8-KI mice. (**A**) Diagram of Psmb8-KI mice. (**B**) Amplification of WT and mutant Psmb8 loci in genomic DNA from WT, Psmb8-KI heterozygous, and Psmb8-KI mice.

To evaluate the mechanism of the inflammatory phenotypes of patients with PRAAS, mice that harbor the human mutation (Gly197Val) in *Psmb8* were established (Figure 1A). We hereafter refer to this mouse strain as the Psmb8–knock-in (Psmb8-KI) mouse strain. The mutation in the *Psmb8* locus in Psmb8-KI mice was confirmed by PCR (Figure 1B).

**Figure 2:**
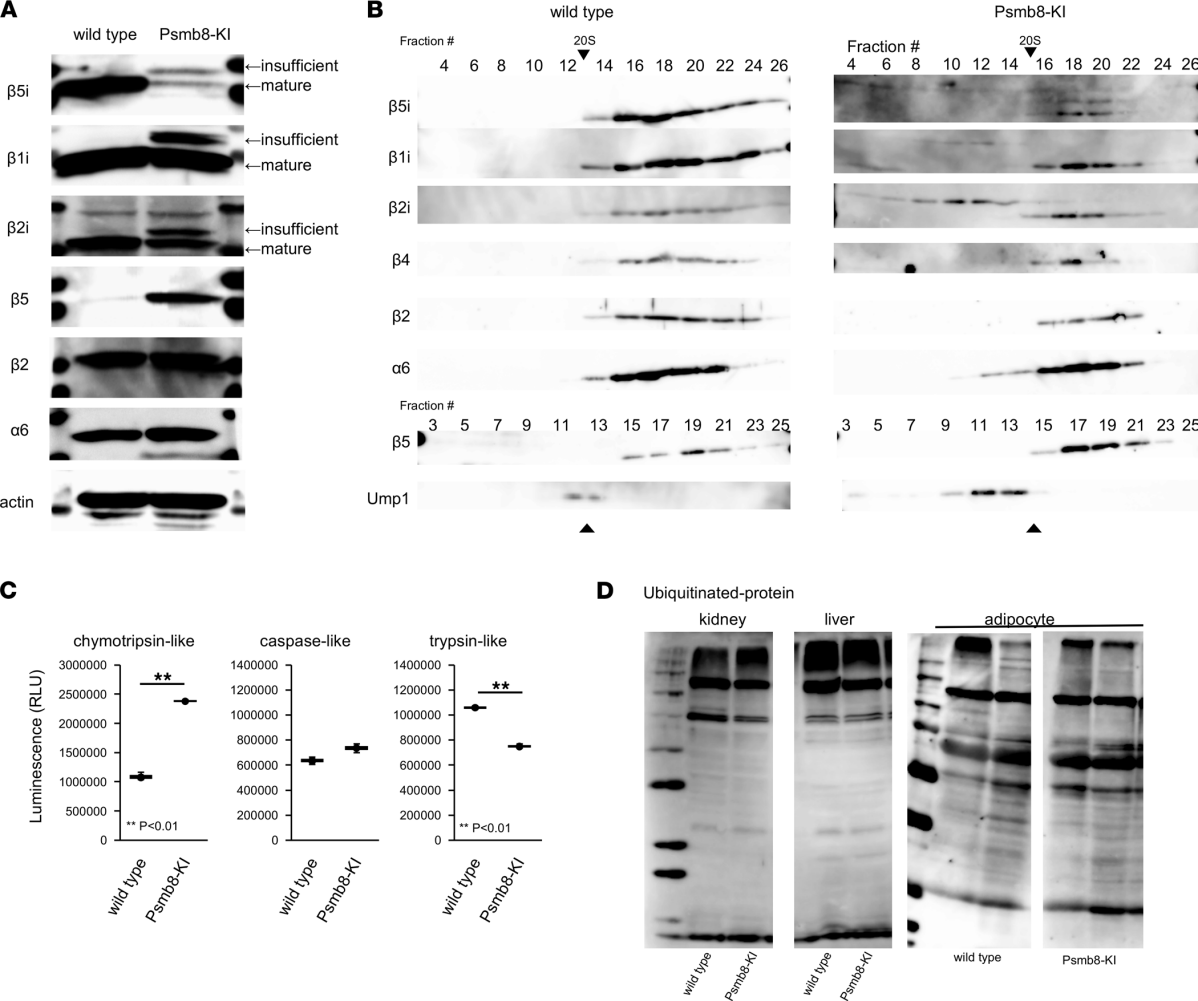
Proteasome assembly and activity of Psmb8-KI mice. (**A**) Spleen cell lysates from WT and Psmb8-KI mice (12-week-old female) were blotted with anti-β5i, anti-β1i, anti-β2i, anti-β5, anti-β2, anti-α6, and anti-actin antibodies. (**B**) Spleen cell lysates from WT and Psmb8-KI mice were separated by glycerol gradient centrifugation. Each fraction was blotted with anti-β5i, anti-β1i, anti-β2i, anti-β4, anti-β2, anti-α6, anti-β5, or anti-Ump1 antibodies. (**C**) The chymotrypsin-, trypsin-, and caspase-like activities of spleen cells from WT and Psmb8-KI mice were measured. Data represent the mean ± SD (*n* = 4 in each group). **P < 0.01(2-tailed unpaired *t* test). (**D**) Cell lysates generated from kidney, liver, and adipose tissues from WT and Psmb8-KI mice were blotted with anti-ubiquitin antibodies. The data in this figure are representatives of 3 independent experiments.

The expression of each immunoproteasome subunit in total spleen cells was evaluated by Western blotting (Figure 2A). The expression levels of mature β5i were reduced, accompanied by insufficiently cleaved β5i, in Psmb8-KI mice compared with WT mice (Figure 2A). Insufficiently cleaved β1i and β2i subunits were also detected in Psmb8-KI mice. The β5 expression was much higher in Psmb8-KI mice than in control mice (Figure 2A). Spleen cell lysates generated from control and Psmb8-KI mice were separated by glycerol gradient centrifugation to determine the mode of immunoproteasome assembly because patients with PRAAS show assembly defects in immunoproteasomes (12). Assembly intermediates containing immature β1i, β2i, and β5i were detected in cell lysates from Psmb8-KI mice, indicating that disturbed assembly of immunoproteasomes was present in these mice, similar to patients with PRAAS with a mutation in *PSMB8* (Figure 2B).

The proteasome activity of total spleen cells from control and Psmb8-KI mice was measured (Figure 2C). Trypsin-like activity was lower in Psmb8-KI mice, while chymotrypsin-like activity was increased in Psmb8-KI mice. Caspase-like activity was comparable between control and Psmb8-KI mice. The ubiquitin expression in Psmb8-KI mouse kidney, liver, and adipose tissues was equivalent to that in corresponding control mouse tissues (Figure 2D).

### Unimpaired immune cell development in Psmb8-KI mice.

**Figure 3:**
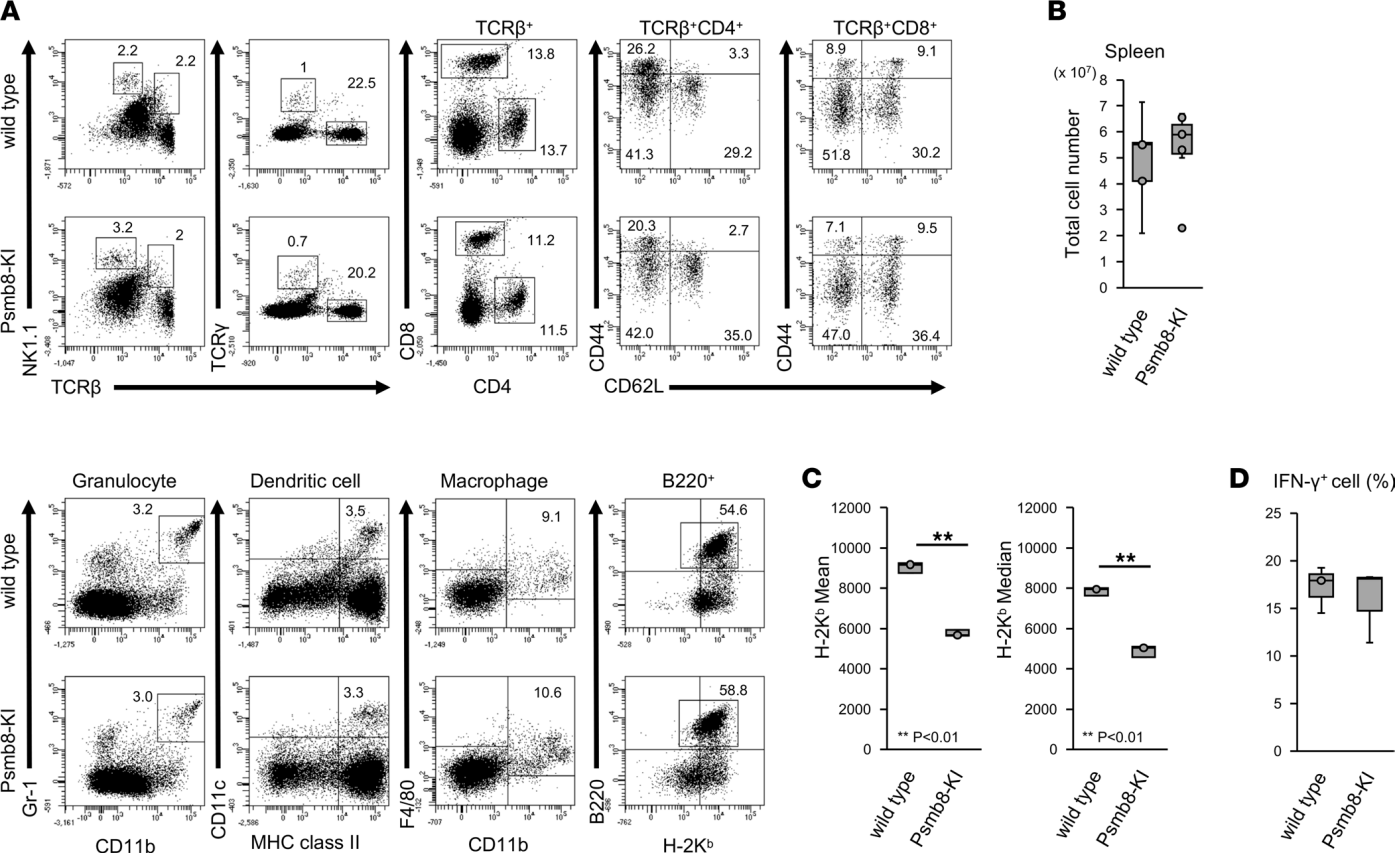
Unimpaired immune cell development in Psmb8-KI mice. (**A**) Spleen cells from WT and Psmb8-KI mice were stained with various combinations of antibodies. (**B**) Total cell number of spleen cells in control and Psmb8-KI mice. Data represent the mean ± SD. *n* = 5. (**C**) The mean fluorescence intensity in K^b+^ cells was measured. Data represent the mean ± SD (*n* = 4 in each group). **P < 0.01 (2-tailed unpaired *t* test). (**D**) Spleen cells from WT and Psmb8-KI mice were stimulated with anti-CD3 and anti-CD28 antibodies for 3 days, and the secretion of IFN-γ was evaluated 5 hours after stimulation with PMA and ionomycin. Data represent the mean ± SD of technical triplicates. The data in this figure are representatives of 3 independent experiments.

The development of immune cells in the spleen was evaluated (Figure 3A). The frequencies of TCRβ^+^, TCRβ^–^NK1.1^+^, B220^+^, CD11b^+^Gr-1^+^, CD11c^+^MHC class II^+^, and CD11b^+^F4/80^+^ cells; the CD4/CD8 ratio in TCRβ^+^ cells; and the CD44/CD62L ratio in CD4^+^ or CD8^+^ cells were comparable between control and Psmb8-KI mice. The total cell number of spleen cells was comparable between control and Psmb8-KI mice (Figure 3B). The expression of H-2K^b^ was reduced (Figure 3, A and C) in Psmb8-KI mice, similar to the expression in *Psmb8*-deficient mice (9). The secretion of IFN-γ by CD4^+^ T cells from Psmb8-KI mice after stimulation with anti-CD3 and anti-CD28 antibodies was equivalent to that by control T cells (Figure 3D).

### Reduced adipose tissue weight in Psmb8-KI mice.

**Figure 4:**
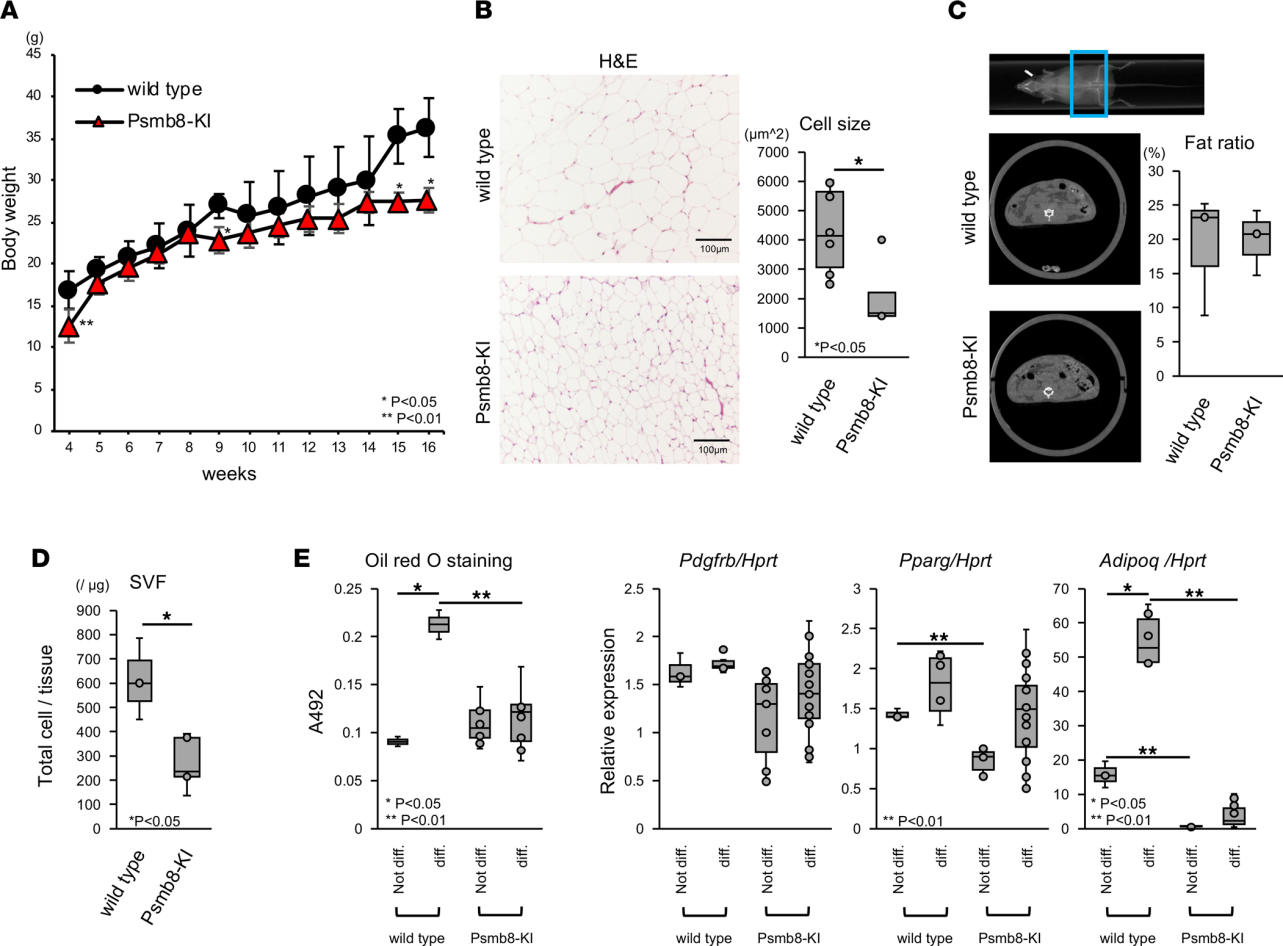
Reduced adipocyte size and fat volume in Psmb8-KI mice. (**A** and **B**) The body weight gain (female WT, closed circle; female Psmb8-KI, red triangle) and size of adipocytes of WT and Psmb8-KI mice were evaluated at 15 weeks old. Scale bar: 100 μm. (**C**) The fat ratio in the total body at the age of 15 weeks was evaluated by CT. Data represent the mean ± SD of technical triplicates. *n* = 5. *P < 0.05 (2-tailed unpaired *t* test). Blue box indicates the region for sagital image. (**D**) The SVF numbers of the adipose tissues of WT and Psmb8-KI mice at 15 weeks old were counted. Data represent the mean ± SD (*n* = 4 in each group). *P < 0.05 (2-tailed unpaired *t* test). (**E**) SVF cells from WT and Psmb8-KI mice were allowed to differentiate into mature adipocytes. Oil Red O staining was performed, and the expression of *Pdgfrb*, *Pparg*, and *Adipoq* was measured by real-time PCR. Data represent the mean ± SD of technical triplicates. *n* = 3. *P < 0.05; **P < 0.01 (1-way ANOVA). The data in this figure are representatives of 3 independent experiments.

Since patients with PRAAS develop partial lipodystrophy and *Psmb8*-deficient mice show reduced adipose tissue weight with high-fat diet feeding (17), we fed control and Psmb8-KI mice a normal diet and measured their body weight from 4–16 weeks old (Figure 4A). The body weight of Psmb8-KI mice was much lower than that of control mice, especially at 15 or 16 weeks of age. Histological examinations of adipose tissue showed smaller adipocytes in Psmb8-KI mice (Figure 4B), and the fat ratio in the whole body tended to be lower in Psmb8-KI mice, as evaluated by computed tomography (Figure 4C).

Psmb8-deficient mice show slower weight gain than WT mice, with reduced adipose tissue volume and smaller mature adipocytes (17). The stromal vascular fraction (SVF) was smaller (Figure 4D), and the differentiation toward adipocytes in the SVF fraction was lower in Psmb8-KI mice than in control mice, as evaluated by Oil Red O staining (Figure 4E); these changes were accompanied by decreased expression of *Pparg* and *Adipoq* (Figure 4E), which encode gene products required for adipocyte differentiation. *Pdgfrb* is expressed in maturation process from the preadipocyte stage to mature adipocytes. Before and after the differentiation, its expression level was comparable between control and Psmb8-KI mice.

### Increased sensitivity to IMS in Psmb8-KI mice.

**Figure 5:**
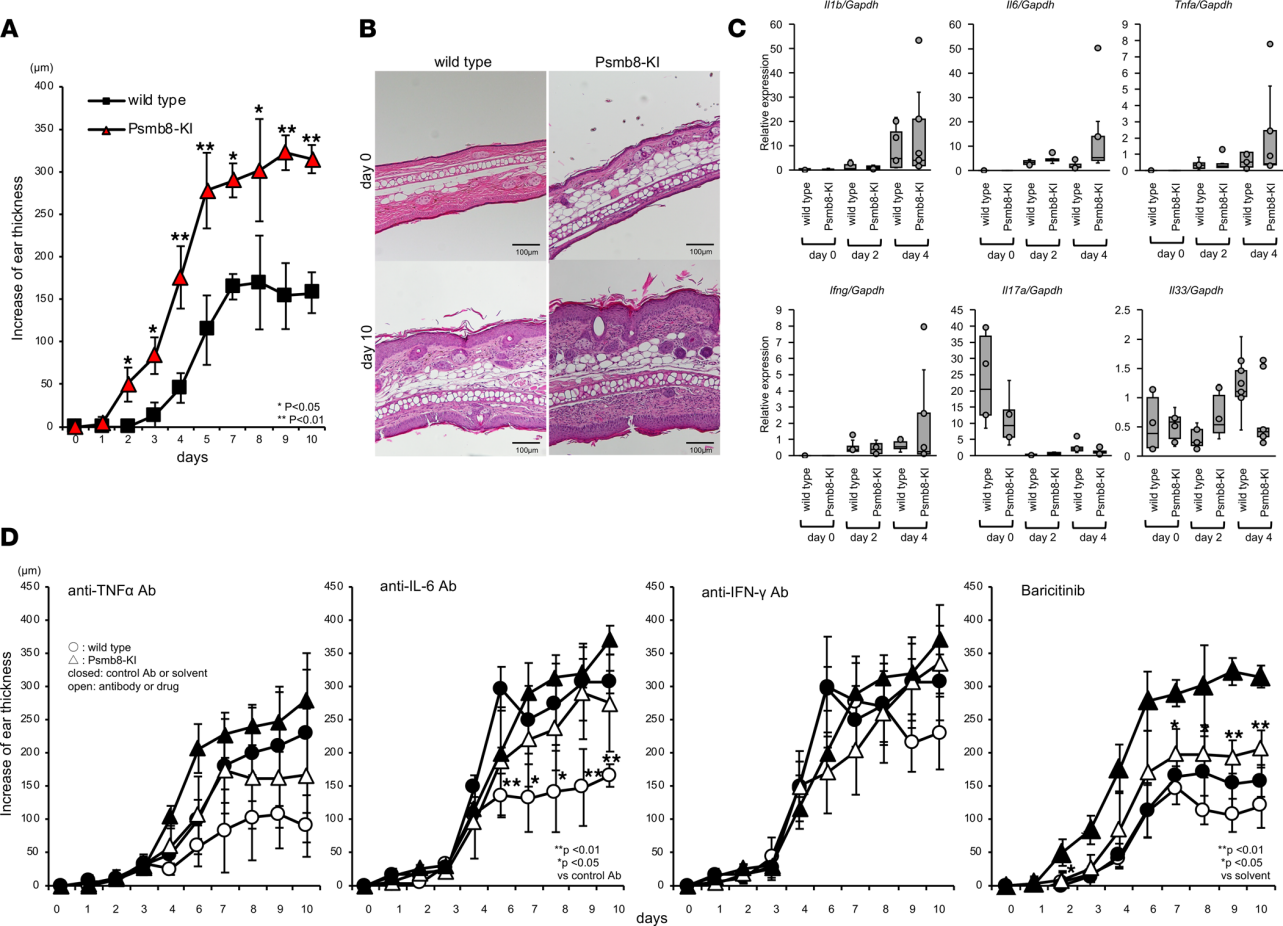
Increased susceptibility to imiquimod-induced dermatitis in Psmb8-KI mice. Imiquimod was applied to the ear skin of WT and Psmb8-KI mice (12- to 15-week-old female mice). (**A**–**C**) The increase in ear thickness (WT, closed square; Psmb8-KI, red triangle) (*n* = 5), histology of the ear skin before and 10 days after imiquimod treatment (HE staining), and expression of proinflammatory genes 10 days after imiquimod treatment were tested (WT, gray; Psmb8-KI, black). Data represent the mean ± SD of technical triplicates. *P < 0.05; **P < 0.01 (2-tailed unpaired *t* test). Scale bars: 100 μm. (**D**) Imiquimod was applied to the ear skin of WT and Psmb8-KI mice, which were treated with anti–IL-6, anti–IFN-γ, or anti–TNF-α antibodies or a JAK inhibitor. WT and Psmb8-KI mice are shown as circles and triangles, respectively. The mice treated with the antibodies or JAK inhibitor are depicted as open symbols. The increase in ear thickness was measured. Data represent the mean ± SD (*n* = 5 in each group). *P < 0.05; **P < 0.01 (1-way ANOVA). The data in this figure are representatives of 5 independent experiments.

As we did not detect any signs of spontaneous inflammation in Psmb8-KI mice even after 6 months of age (data not shown), we tested the sensitivity to imiquimod-induced dermatitis (IMS). Imiquimod was painted on the ear skin, and ear thickness was measured (Figure 5A). We began to measure ear thickness in Psmb8-KI mice 2 days after the initial treatment, and increased ear thickness was found in Psmb8-KI mice from 2 to 10 days after the initial treatment. Histological studies performed 10 days after the initial treatment demonstrated a thicker ear and more infiltration of mononuclear cells in the skin in Psmb8-KI mice than in WT mice (Figure 5B). Since imiquimod is an agonist of TLR7, TLR7-associated inflammatory genes were evaluated by real-time PCR (Figure 5C). The expression of *Il6*, *Ifng*, and *Tnfa* in regions treated with imiquimod tended to be higher in Psmb8-KI mice 4 days after the initial treatment.

We administered anti–IL-6, anti–TNF-α, or anti–IFN-γ antibodies or baricitinib (a JAK1/2 inhibitor) to mice administered imiquimod on the ear skin (Figure 5D). The anti–TNF-α and anti–IL-6 antibodies ameliorated ear thickening in both control mice and Psmb8-KI mice, but a difference in ear thickness was still detected between the control and Psmb8-KI mice treated with either antibody. In contrast, anti–IFN-γ treatment did not ameliorate IMS in either control mice or Psmb8-KI mice. Treatment with baricitinib most significantly reduced ear thickness in both control mice and Psmb8-KI mice, but a difference in ear thickness was still detected between the control and Psmb8-KI mice treated with baricitinib. These data indicate that blockade of IL-6 or TNF-α, or treatment with baricitinib, can suppress ear inflammation, while the magnitude of the suppressive effect is similar in control and Psmb8-KI mice, suggesting that other mediators are responsible for the higher sensitivity to inflammation in Psmb8-KI mice.

### Cxcl10 is highly expressed following proteasome inhibition or in Psmb8-KI mice.

**Figure 6:**
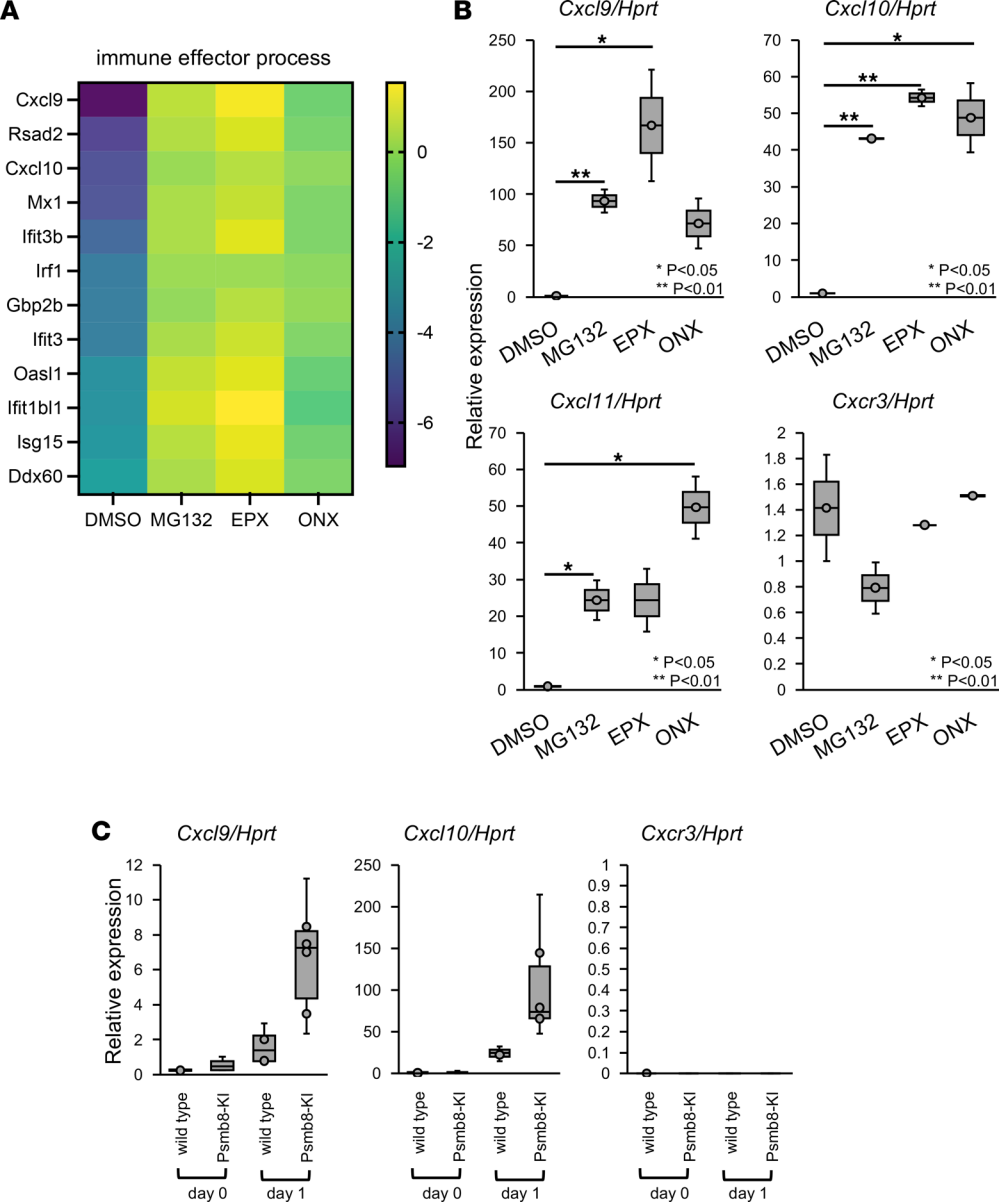
Increased expression of Cxcl9 and Cxcl10 induced by inhibiting proteasomes. (**A** and **B**) J774 cells were treated with MG132, epoxomicin, and ONX0914 for 4 hours, and gene expression was evaluated with DNA microarray and real-time PCR. Differences in each gene were classified by the color in **A**. Data represent the mean ± SD of technical triplicates. *P < 0.05; **P < 0.01 (1-way ANOVA). (**C**) The expression of genes in skins that were painted with imiquimod from WT and Psmb8-KI mice was measured by real-time PCR (WT, gray; Psmb8-KI, black). The data in this figure are representatives of 3 independent experiments.

To identify the molecules that underlie the differential sensitivity to IMS between control and Psmb8-KI mice, we compared genes that were differentially expressed following treatment with proteasome inhibitors (Figure 6A). We treated J774 cells with 1 of 3 proteasome inhibitors — MG132, epoxomicin, or ONX0914 — for 4 hours and tested gene expression patterns with a DNA microarray. The data was submitted to the NCBI Gene Expression Omnibus (GEO) database (https://www.ncbi.nlm.nih.gov/geo/; accession no. GSE189308). Among immune-associated genes, the chemokines *Cxcl9* and *Cxcl10*, which use the receptor CXCR3, exhibited higher expression in Psmb8-KI mice than in control mice (Figure 6A). The higher expression of *Cxcl9* and *Cxcl10*, as well as elevated expression of *Cxcl11*, was confirmed by real-time PCR, while the expression of their receptor, *Cxcr3*, was not increased (Figure 6B). Spleen cells from Psmb8-KI mice also highly expressed *Cxcl9* and *Cxcl10* compared with control cells (Figure 6C). These data suggest that the CXCR3 pathway is activated in Psmb8-KI mice.

### Deficiency in Cxcr3 ameliorated IMS in Psmb8-KI mice.

**Figure 7:**
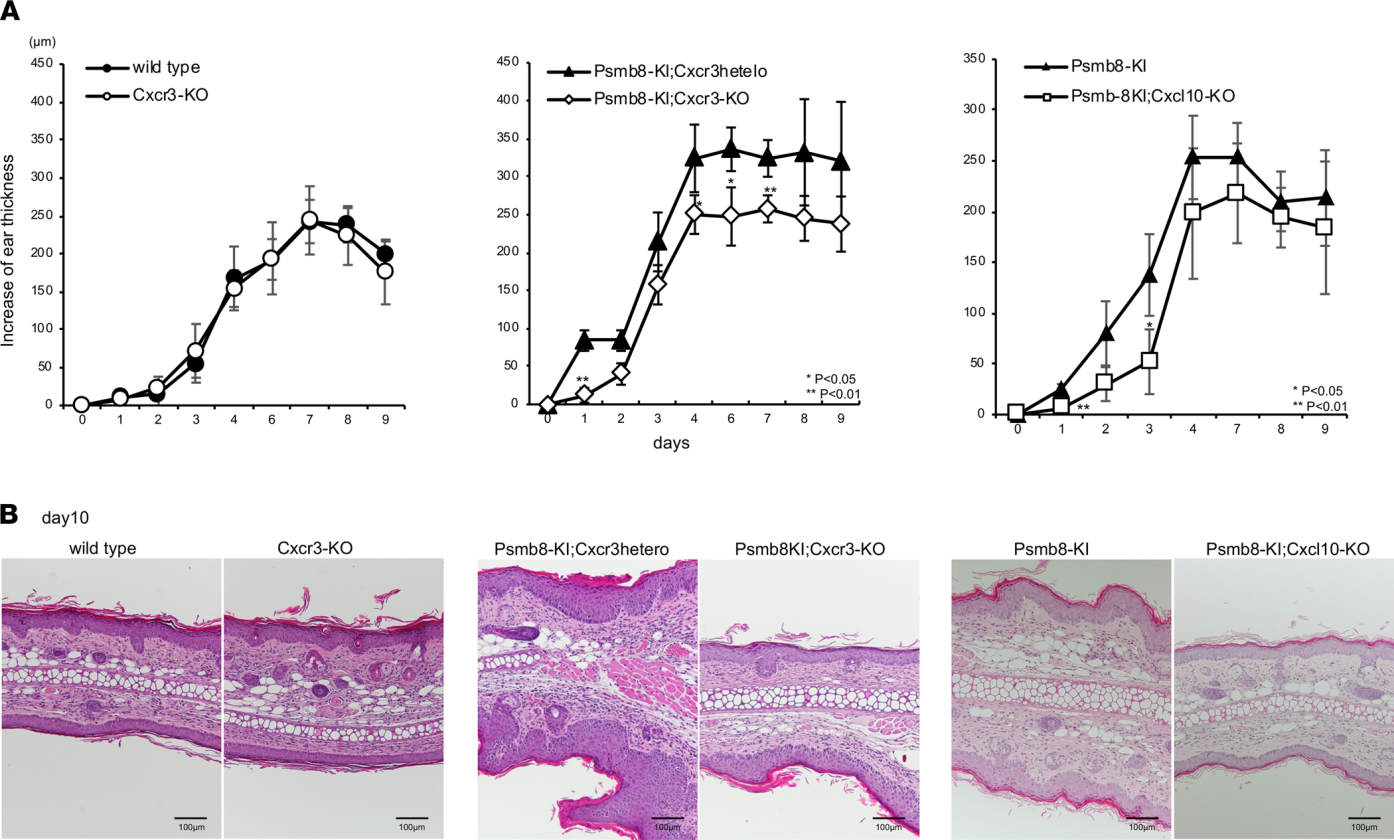
Deficiency in the Cxcr3 gene in Psmb8-KI mice decreased IMS. Imiquimod was applied to the ear skin of WT (open circle) and Cxcr3-deficient (closed circle) mice, Psmb8-KI (closed triangle) and Psmb8-KI;Cxcr3-KO (open square) mice, or Psmb8-KI (closed triangle) and Psmb8-KI;Cxcl10-KO (open square) mice. (**A** and **B**) Increased ear thickness (*n* = 5 in each group) and ear histology were tested. All groups used 12- to 15-week-old female mice. Data represent the mean ± SD of technical triplicates. *P < 0.05; **P < 0.01 (2-tailed unpaired *t* test). The data in this figure are representatives of 3 independent experiments.

We sought to analyze the contribution of CXCR3 to the higher sensitivity to IMS in Psmb8-KI mice. We first applied imiquimod to the ear skin of WT and *Cxcr3*-deficient mice (Figure 7A). The increase in ear thickness seen in the *Cxcr3*-deficient mice was equivalent to that observed in the control mice, suggesting that CXCR3 is not involved in the disease progression of IMS in control mice. We then applied imiquimod to the ear skin of Psmb8-KI mice deficient in the *Cxcr3* or *Cxcl10* gene (Psmb8-KI;CXCR3-KO or Psmb8-KI;CXCL10-KO) (Figure 7, A and B). Ear thickening was suppressed in the absence of *Cxcr3* and tended to be inhibited in the absence of *Cxcl10* on the Psmb8-KI background. These data demonstrate that the CXCR3 pathway is the key signaling pathway underlying the increased susceptibility to IMS in Psmb8-KI mice.

## Discussion

Patients with PRAAS exhibit inflammatory signs in various organs (12, 18, 19). Although those inflammatory symptoms are, at least partially, improved by treatment with a JAK1/2 inhibitor (16, 20), the molecular mechanisms by which these inflammatory responses are induced and which molecules are key in the initiation or progression of inflammation in patients with PRAAS are unknown. Here, we demonstrated that the CXCR3 pathway is hyperactivated in Psmb8-KI mice and that deficiency in the *Cxcr3* gene in Psmb8-KI mice ameliorates IMS. These data suggest the CXCR3 pathway as a target for treating patients with PRAAS.

Psmb8-KI mice on a C57BL/6 background did not exhibit any inflammatory signs, even after 6 months of age, in our specific pathogen–free (SPF) facility (data not shown). In contrast to the accumulation of ubiquitin-coupled proteins in the cells of patients with PRAAS (12), the total spleen cells of Psmb8-KI mice did not show increased accumulation of ubiquitin, although the patterns of chymotrypsin, trypsin, and caspase activities were distinct between control and Psmb8-KI mice. One possible reason for why Psmb8-KI cells did not show accumulation of ubiquitin-coupled proteins is the overexpression of β5 in Psmb8-KI mice, as our previous experiments using PRAAS cells did not show overexpression of β5 (12). This overexpression of β5 might, at least partially, compensate for the immunoproteasome dysfunction caused by the mutation in β5i, allowing cells to degrade ubiquitin-coupled proteins in a manner almost equivalent to that of control cells. Another point that needs to be discussed is the distinct patterns of chymotrypsin, trypsin, and caspase-like activities between control and Psmb8-KI mice. Given the high chymotrypsin activity and low caspase-like activity of immunoproteasomes compared with those of constitutive proteasomes, the increased chymotrypsin activity and reduced trypsin-like activity in Psmb8-KI cells compared with control cells suggest that an assembly defect in immunoproteasomes dynamically changes the expression of each catalytic subunit per cell. The hyperexpression of β5 might also contribute to the different patterns of protease activity in Psmb8-KI mice.

CXCL9, CXCL10, and CXCL11 are known to be Th1 chemokines and to bind to CXCR3. These chemokines attract Th1 cells into inflamed tissues, where Th1 cells produce cytokines, which leads to increased Th1 chemokines levels in the inflamed tissues and amplification of the feedback loop. The levels of these chemokines are elevated in autoimmune and rheumatic diseases and in cancers (21–25). Several papers have reported that patients with PRAAS exhibit a typical type I IFN signature with increased expression of IFN-stimulated genes, including *CXCL9* and *CXCL10* (15, 26, 27). We here detected increased levels of *Cxcl9* and *Cxcl10* gene expression in J774 cells treated with proteasome inhibitors and spleen cells from Psmb8-KI mice, suggesting that elevated levels of CXCL9 and CXCL10 hyperactivate CXCR3 in Psmb8-KI mice. Indeed, the suppression of inflammation in Psmb8-KI mice induced by deficiency in *Cxcr3* gene expression reveals that activation of the CXCR3 pathway is one of the key events leading to the increased susceptibility to IMS in Psmb8-KI mice. What is the mechanism underlying the upregulation of CXCL9 and CXCL10 induced by a mutation in *Psmb8*? The J774 cells treated with proteasome inhibitors did not upregulate type I or type II IFNs (data not shown), suggesting that the upregulation of CXCL9 and CXCL10 induced by inhibiting proteasomes could be attributed to cell-intrinsic regulation of both genes. Previous studies using human cells also found that treatment of peripheral mononuclear cells and fibroblasts upregulated mRNA of *CXCL10* but not mRNA of proinflammatory cytokines, including *IL6* and *IL1B* (15). Therefore, although increased expression of CXCL10 in patients with PRAAS might occur in response to various inflammatory stimuli, increased expression of those chemokines could be regulated, at least partly, in a cell-intrinsic manner.

Patients with PRAAS are characterized by hypergammaglobulinemia, and some patients showed low CD8^+^ T cell counts and low percentages of naive CD8^+^ T cells (20, 28). Since immunoproteasomes are involved in generating peptides presented by MHC class I (7), patients with PRAAS might have a distinct repertoire of CD8^+^ T cell receptors compared with healthy people, which might contribute to inflammatory responses in patients with PRAAS. In addition, CXCR3 is highly expressed on mouse and human T cells and functions as the migration of T cells into inflammatory regions (21). The suppression of inflammation in Psmb8-KI mice by blockade of CXCR3 in this study would also suggest the contributions of CD8^+^ T cells to the inflammation in patients with PRAAS.

The mutation of *PSMB8* was originally identified in patients with PRAAS (12–14), and subsequent studies reported that mutations in other proteasome subunits also led to PRAAS phenotypes (15). Recent studies have reported that the mutation in *PSMB9* causes immunodeficiency phenotypes (29). Therefore, it would be important to evaluate the relationship between the altered function of each proteasome subunit and the associated mutation. Here, we used 3 proteasome inhibitors with distinct specificities: (a) MG132, an inhibitor of chymotrypsin; (b) epoxomycin, a 20S proteasome inhibitor; and (c) ONX0914, a β5/β5i inhibitor. The data show the treatment with any 3 inhibitors upregulated *Cxcl9* and *Cxcl10*. In addition to revealing the mechanism by which altered function of proteasomes upregulated those chemokines, it would be interesting to analyze the function of each subunit of the proteasomes and chemokine expression in future studies.

The CXCR3 pathway has been a drug target of interest in inflammatory disorders (30, 31). Indeed, a clinical trial was performed to evaluated rheumatoid arthritis treatment with an anti-CXCL10 blocking antibody (31), which showed partial improvement of inflammatory responses. Since our present study showed the involvement of the CXCR3 pathway in increased susceptibility to inflammation, antagonists of CXCR3 or monoclonal antibodies against CXCL10 might be effective for treating patients with PRAAS. However, as we have shown the increased susceptibility of Psmb8-KI mice to IMS, we need to establish an animal model that spontaneously reproduces inflammatory responses similar to those of patients with PRAAS. In addition, in this study, we used *Cxcl10*-deficient mice from a C57BL/6 strain that has a frameshift mutation in the *Cxcl11* gene (32). CXCL11 is a strong inducer of CXCR3 internalization (33) that may lead to reduced accessibility of CXCL9 and CXCL10 to CXCR3. Thus, we need to reanalyze the effect of CXCL10 in a different background in future studies.

Psmb8-KI mice showed a reduced volume of adipose tissue and impaired differentiation of mature adipocytes, similar to *Psmb8*-deficient mice (17). The accumulation of ubiquitin-coupled proteins was comparable between control and Psmb8-KI mice, although the patterns of proteasome activity were altered in Psmb8-KI mice. Therefore, the impaired adipocyte differentiation in Psmb8-KI mice could likely be attributed to the change in the turnover of particular proteins — not only cell stress or death caused by the accumulation of ubiquitin-coupled proteins.

In conclusion, we identified the CXCR3 pathway as a drug target in PRAAS. Treatment with a JAK1/2 inhibitor led to therapeutic efficacy limiting inflammatory responses, although some patients still exhibit residual inflammation with this approach. Thus, potent CXCR3 inhibitors might have additive therapeutic potential when combined with a JAK1/2 inhibitor to treat patients with PRAAS. Finally, it should be noted that it remains unclear how CXCL9 and CXCL10 expression is increased and how the IFN signature is induced in patients with PRAAS. The elucidation of the steps involved in the initiation of inflammation by immunoproteasome dysfunction would help identify additional disease-specific therapeutic targets in PRAAS.

## Methods

### Mice.

C57BL/6 mice were purchased from Charles River Laboratories Japan. Cxcr3^–/–^ and Cxcl10^–/–^ mice were purchased from The Jackson Laboratory. Psmb8-KI mice were established by constructing a targeting vector with a mutation in Psmb8. Psmb8-KI mouse genotyping was performed with tail-derived DNA by genomic PCR (primer 1: 5′-ccgaggtgtcttacccattgaacc-3′, primer 2: 5′-ttgccgaatatcatggtggaaaatggc-3′, and primer 3: 5′-agagatggctcagtggttaagaccc-3′). All mice were maintained in the animal research center of Tokushima University.

### Protein extracts, Western blotting, and glycerol gradient analysis.

Splenocytes were lysed in RIPA buffer (Nakalai Tesque) containing protease inhibitors (Roche). The lysates were resolved by SDS-PAGE, and the blots were incubated with anti-β1i, anti-β2, anti-β2i (provided by Shigeo Murata, The University of Tokyo, Tokyo, Japan.), anti-α6 (ab109530), anti-ubiquitin (ab7780, Abcam), anti-β4 (PW8899, Enzo Life Sciences), anti-β5 (PW8895, Enzo Life Sciences), anti-β5i (PTG14859, Proteintech), anti–Ump-1 (15046-1-AP, Proteintech), or anti-actin (A2066, Sigma-Aldrich) antibodies. Then, the blots were incubated with HRP-conjugated goat anti–rabbit IgG antibodies (170–6515; Bio-Rad). The bands were detected with ECL Prime Chemiluminescent Substrate (GE Healthcare) and an ImageQuant LAS-4000 mini system (GE Healthcare). For glycerol gradient analysis, cell lysates were fractionated by linear glycerol density gradient centrifugation (22 hours, 100,000*g*, 4°C) as described previously (12).

### Proteasome activity assay.

Splenocytes were assayed using the Proteasome-Glo Cell-Based Assay (Promega), according to the manufacturer’s protocol.

### Flow cytometry.

Splenocytes were incubated with 2.4G2 supernatant to block Fc receptors. Then, the cells were incubated with fluorochrome-conjugated monoclonal antibodies specific for mouse TCRβ (clone H57-597), TCRγ (clone GL3), NK1.1 (clone PK136), CD4 (clone GK1.5), CD8 (clone 53–6.7), CD44 (clone IM-7), CD62L (clone MEL-14), Gr-1 (clone RB6-8C5), CD11b (clone M1/70), CD11c (clone N418), I-A (clone M5/114.15.2), F4/80 (clone BM8), B220 (clone RA3-6B2), and H-2K^b^ (clone 28-8-6) (BioLegend). Data were collected on a FACS Canto II flow cytometer (BD Biosciences) and analyzed using FACSDiva (BD Biosciences).

Whole splenocytes (1 × 10^6^/well; 48-well plates) were cultured with a plate-coated anti-CD3 monoclonal antibody (1 μg/mL) (145-2C11, BioLegend) for 72 hours. PMA (25 ng/mL), ionomycin (1 μg/mL) (BioLegend), and monensin (2 μM) (ab193381, Abcam) were added to the cell culture medium for 4 hours prior to harvest. For intracellular staining, cells were fixed with 4% paraformaldehyde, permeabilized using 90% methanol, and stained with fluorochrome-conjugated monoclonal antibodies specific for mouse anti–IFN-γ (XMG1.2, BioLegend)

### Real-time PCR.

RNA was extracted from cells or imiquimod-treated (IMQ-treated) ears, with degradation to genomic DNA, using ReliaPrep RNA Cell Miniprep Systems or ReliaPrep RNA Tissue Miniprep Systems (Promega). cDNA was reverse transcribed with ReverTra Ace quantitative PCR RT Master Mix with gDNA Remover (TOYOBO). PCR analyses were performed using StepOnePlus (Applied Biosystems) with primers.

### Histology.

Epididymal adipose tissues and ear skin samples were collected and fixed in a 10% formalin solution. Paraffin-embedded tissue samples were sectioned and stained with H&E.

### Adipocyte differentiation assay.

Epididymal adipose tissues were cut into small pieces, followed by incubation in adipose isolation buffer (17) containing 1 mg/mL collagenase (Wako Pure Chemical Industries) for 1 hour at 37°C with gentle shaking. SVF cells were collected as a pellet by centrifugation at 500*g* and 4°C for 5 minutes. SVF cells were cultured in 10% FBS-DMEM supplemented with a penicillin/streptomycin solution (Thermo Fisher Scientific). Two days after reaching confluence, cells were incubated in differentiation medium (AdipoInducer Reagent [for animal cells]; Takara Bio Inc.) containing dexamethasone (2.5 μM), 3-isobutyl-1-methylxanthine (0.5 mM), and insulin (10 μg/mL) (included AdipoInducer Reagent [for animal cells]; Takara Bio Inc.) for 2 days. The medium was then replaced with maintenance medium (insulin [10 μg/mL] in 10% FBS-DMEM supplemented with antibiotics). The maintenance medium was renewed every 2 or 3 days for 6 days of culture. Adipocyte differentiation was evaluated by Oil Red O staining. Oil Red O uptake into cells was quantified by extraction with isopropanol, and the absorbance of the eluate was measured at 492 nm.

### μ-CT.

The region comprising the 12th thoracic vertebra to the 4th sacral vertebra in mice was analyzed in vivo with μ-CT at high resolution. The weight of fat tissue was calculated using Latheta software (Latheta LCT-200, Hitachi Aloka Medical).

### IMS model.

Mice received a daily topical dose of 25 mg of commercially available imiquimod cream (5%) (Beselna Cream, Mochida Pharmaceutical) on the right ear for 10 days, with the exception of day 5 or 6. For blockade experiments, a control antibody or an anti–IL-6 (MP5-20F3), anti–TNF-α (XT3.11), or anti–IFN-γ (XMG1.2) monoclonal antibody (BioXCell) was administered on 2 consecutive days, followed by no treatment on the third day for 10 days at 400 μg/dose via i.p. injection. Mice received 6 total injections. Baricitinib (AdooQ BioScience) was administered daily by oral gavage at 10 mg/kg/dose for 10 days, with the exception of day 5.

### DNA microarray.

The mouse monocyte-macrophage cell line J774 was cultured in 10% FBS-RPMI medium (Nakalai Tesque) supplemented with a penicillin/streptomycin solution (Thermo Fisher Scientific) in the presence of the proteasome inhibitor MG132 (LifeSensors), the 20S proteasome inhibitor epoxomicin (PEPTIDE INSTITUTE), or the β5/β5i inhibitor ONX 0914 (Adooq Bioscience) at 1 μM or DMSO (Sigma-Aldrich) as a control solvent for 4 hours. RNA was extracted from cultured J774 cells, with genomic DNA degradation, using ReliaPrep RNA Cell Miniprep Systems (Promega). The quality of the isolated RNA was evaluated with an Agilent 2100 BioAnalyzer. Probe preparation and microarray analyses were performed on Whole Human Genome Microarray 4x44K v2 (Agilent Technologies). The resulting data were normalized using GeneSpring (Agilent Technologies) software. The DNA microarray data were submitted to the NCBI GEO database (https://www.ncbi.nlm.nih.gov/geo/; accession no. GSE189308). 

### Data transfer agreements.

The primer sequences are available upon request.

### Statistics.

For all experiments, significant intergroup differences were calculated using 2-tailed unpaired *t* test or 1-way ANOVA. Differences were considered significant when *P* < 0.05.

### Study approval.

All animal experiments were approved by the animal research committee of Tokushima University and performed in accordance with our institution’s guidelines for animal care and use.

## Author contributions

YS and KY designed the research; YS did most of the experiments; HA, ST, HK, and KO analyzed the data; YS and KY wrote the paper; HA, ST, HK, and KO reviewed the paper; and KY supervised all researches.

## Version 1 04/08/2022

Electronic publication
